# Nonlinearly interacting entrainment due to shear and convection in the surface ocean

**DOI:** 10.1038/s41598-022-14098-w

**Published:** 2022-06-14

**Authors:** Yusuke Ushijima, Yutaka Yoshikawa

**Affiliations:** 1 Japan Meteorological Business Support Center, Tsukuba, 305-0052 Japan; 2 Meteorological Research Institute, Tsukuba, 305-0052 Japan; 3 Graduate School of Science, Kyoto University, Kyoto, 606-8502 Japan

**Keywords:** Physical oceanography, Fluid dynamics

## Abstract

Large-eddy simulations were performed to investigate the entrainment buoyancy flux at the mixed layer base due to nonlinearly interacting shear-driven turbulence (ST) and convective turbulence (CT). The fluxes due to pure ST and pure CT were first evaluated, and their scalings were derived. The entrainment flux due to coexisting ST and CT was then evaluated and compared to the scaling-based fluxes due to the pure turbulences. It was found that nonlinear effects reduce the entrainment flux by $30 \%$ when the turbulent kinetic energy production rates of ST and CT are comparable. The mixing parameterization schemes used in ocean general circulation models (OGCMs) fail to accurately reproduce the mixing due to the pure turbulences and/or the nonlinear effects, and thus the mixed layer depth (MLD). Because analysis using global datasets suggests that nonlinear effects are large at the mid-latitudes, these results indicate that the nonlinear effects might be responsible for the deep MLD biases in OGCMs and that mixing parameterization schemes need to be improved to correctly represent ocean surface mixing due to shear and convection, as well as waves, in OGCMs.

## Introduction

Vertical turbulent mixing induced by wind, surface cooling, and surface waves forms vertically uniform surface mixing/mixed layer (ML) in the upper stratified ocean. As the mixing deepens the ML, water below the ML is entrained into the layer, and ML water properties are changed. For example, the entrainment of colder and nutrient richer water changes temperature and the concentration of nutrients in the ML and then affects subsequent air-sea interaction^1,2^ and primary production^3,4^, respectively. It is in fact suggested that the ML deepening due to the entrainment enhances the variability of Pacific Decadal Oscillation and then affects the regional and global climate systems^2^.

In ocean general circulation models (OGCMs), mixing parameterization schemes such as the Mellor–Yamada (MY) scheme^5^ and the K-profile parameterization (KPP) scheme^6^ are adopted to represent entrainment. Because the parameterization of the entrainment buoyancy flux at the ML base ($P_b$) is key to developing the mixing parameterization, the flux has been investigated in several studies^7–10^. Studies of the atmospheric boundary layer (ABL)^11–14^ are also useful because the entrainment process in the ocean surface boundary layer is dynamically similar to that in the ABL. Many ABL studies^15,16^ focused on the entrainment process caused by convective turbulence (CT) because it is likely dominant in the ABL.

In the ocean, CT is considered to be dominant if the sea surface is severely cooled. Deep convection down to $1000 \ \mathrm{m}$ depth and greater in Labrador and Greenland seas is an example. When CT is dominant, temperature, salinity and momentum become almost uniform in the vertical. Under purely convective forcing (no wind forcing), the entrainment buoyancy flux at the ML base ($P_b^{C}$) in the ocean is considered to be proportional to the surface buoyancy flux ($B_f$, which is defined as positive for sea surface cooling) and is expressed as

1 $$\begin{aligned} P_b^{C} = - n B_f. \end{aligned}$$

Here, $n \cong 0.2$^9,13,14^ when convection is shallow. If, on the other hand, convection is deep and turnover time of convection $L_{MLD}/W_*$ is longer than 1/*f*, where $W_* [\equiv (B_fL_{MLD})^{1/3}]$ is convective velocity scale, $L_{MLD}$ is ML depth (MLD), and *f* is the Coriolis parameter, the Earth rotation (the Coriolis acceleration term) acts and inhibits vertical velocity and hence CT^17^. Thus, the Earth’s rotation decreases *n* at the convective Rossby number ${\mathrm{Ro}_\mathrm{b}} \equiv W_*/fL_{MLD} \lesssim 1$^18–20^.

If, on the other hand, the sea surface is weakly cooled as in low-latitude ($< 10^\circ$) regions, wind-induced shear-driven turbulence (ST) becomes dominant. When ST is dominant, temperature and salinity are well homogenized in the vertical while momentum is not, because the turbulent kinetic energy (TKE) in the ML is produced by the vertical shear of the horizontal velocity at each depth. At the ML base, the TKE is converted to the potential energy, and the entrainment buoyancy flux ($P_b^S$) is expressed as

2 $$\begin{aligned} P_b^S = - m \frac{U_*^3}{L_{MLD}}, \end{aligned}$$

where $U_*$ is the friction velocity and *m* is a proportional coefficient^8^. It should be noted that the depth scale of the wind-induced shear is given by $U_*/f$ (turbulent Ekman scale)^21^ and the shear becomes almost zero at the depth below $U_*/f$ in neutrally stratified fluid. This indicates that the shear at the ML base is weakened (intensified) if $L_{MLD}$ is greater (smaller) than $U_*/f$. Consequently, *m* in Eq. (2) should depend on the Rossby number $\mathrm{Ro} \equiv U_*/fL_{MLD}$^10,22^. However, the dependence of *m* on $\mathrm{Ro}$ has not been well investigated.

In autumn and winter, especially at mid-latitudes, ST and CT typically coexist in the ML. Some previous studies^9,10,23^ assumed for simplicity that the entrainment buoyancy flux at the ML base due to coexisting ST and CT ($P_b$) can be expressed as the linear combination of the entrainment buoyancy fluxes due to pure ST and pure CT, that is,

3 $$\begin{aligned} P_b = P_b^S + P_b^C. \end{aligned}$$

However, previous ABL studies showed that the ABL structure with coexisting ST and CT is different from the structure with each pure turbulence^24–26^, and thus CT is suppressed in the entrainment zone (corresponding to the ML base in the ocean) by ST^14^. These results indicate that the effects of nonlinear interaction between ST and CT may also be large in the surface ocean, and thus the entrainment buoyancy flux $P_b$ at the ML base cannot be expressed as a linear combination of $P_b^S$ and $P_b^C$. Nevertheless, the nonlinear effects were not evaluated under realistic ocean surface forcing, and it is not clear when and where the nonlinear effects, if any, are large.

It is well known that recent OGCMs still have serious biases in simulating MLDs^27^. In recent decades, much attention has been directed to the shallow MLD biases in OGCMs and the effects of surface waves on these biases^27,28^. Surface waves interact with the wind-driven flow shear to form secondary circulations (Langmuir circulations) that induce turbulence and deepen the ML^29–31^. Non-breaking surface waves without wind-driven flow may also cause turbulence and deepen the ML^32,33^. Most OGCMs do not include these surface wave effects, and this is considered as one of the main reasons for the shallow MLD biases. Note, however, that deep MLD biases have also been found; the MLDs in OGCMs are sometimes greater than the observed values in regions such as the mid-latitudes (see Fig. 1 of Belcher et al.^28^ and Fig. 11 of Tsujino et al.^34^), even though most OGCMs omit surface wave effects. This fact clearly demonstrates that entrainment processes due to ST and CT also need to be re-investigated quantitatively.

The aim of this study is to evaluate the entrainment buoyancy flux at the ML base induced by nonlinearly interacting ST and CT and quantify the nonlinear effects in the surface ocean. Surface wave effects are not considered here to isolate this interaction processes from other complicated processes. To this end, large-eddy simulations (LESs) of the upper-ocean turbulence forced by uniform steady wind stress and/or cooling were performed; the configuration is described in "Methods" section. Uniform and steady surface forcing is used as a first step to understand nonlinear interaction between ST and CT. In "Results" section, we first evaluated the parameter dependences of the entrainment buoyancy flux due to pure ST and pure CT and derived the scaling of each type of turbulence. Then, we quantified the nonlinear effects by comparing the entrainment flux due to coexisting ST and CT with the fluxes due to each pure turbulence under the realistic ocean forcing parameters. The mixing parameterization schemes used in OGCMs were also tested to see whether the entrainment buoyancy flux due to nonlinear effects as well as the pure turbulence is reproduced. The global distribution of the intensity of the nonlinearity is presented using global datasets in "Discussion" section.

## Results

In this section, we first show simulated profiles of horizontally averaged flow, buoyancy, and TKE tendency terms in typical cases. Then, we evaluate the simulated entrainment buoyancy flux due to pure ST and pure CT to obtain their scalings ( $P_b^S$ and $P_b^C$, respectively). Using the scalings, we evaluate the simulated entrainment buoyancy flux due to coexisting ST and CT ($P_b$) and compare $P_b$ with $P_b^S+P_b^C$ to quantify the nonlinear interaction effects between ST and CT. Finally, the mixing parameterization schemes are tested to evaluate the extent to which they reproduce the entrainment flux of the pure turbulences and the nonlinear effects.

### Profiles of horizontally averaged velocity, buoyancy, and TKE tendency terms in the ML: Typical cases

First, the results of typical cases of pure ST, pure CT, and coexisting ST and CT are shown. In this subsection, the initial stratification ($N_0 = 2.0 \times 10^{-2} \, {\mathrm{s}^{\mathrm{-1}}}$), initial MLD ($L_0 = L_D/4$, where $L_D$ is the domain size described in "Methods" section), and Coriolis parameter ($f = 10 \times 10^{-5} \, {\mathrm{s}^{\mathrm{-1}}}$) are unchanged.

**Figure 1:**
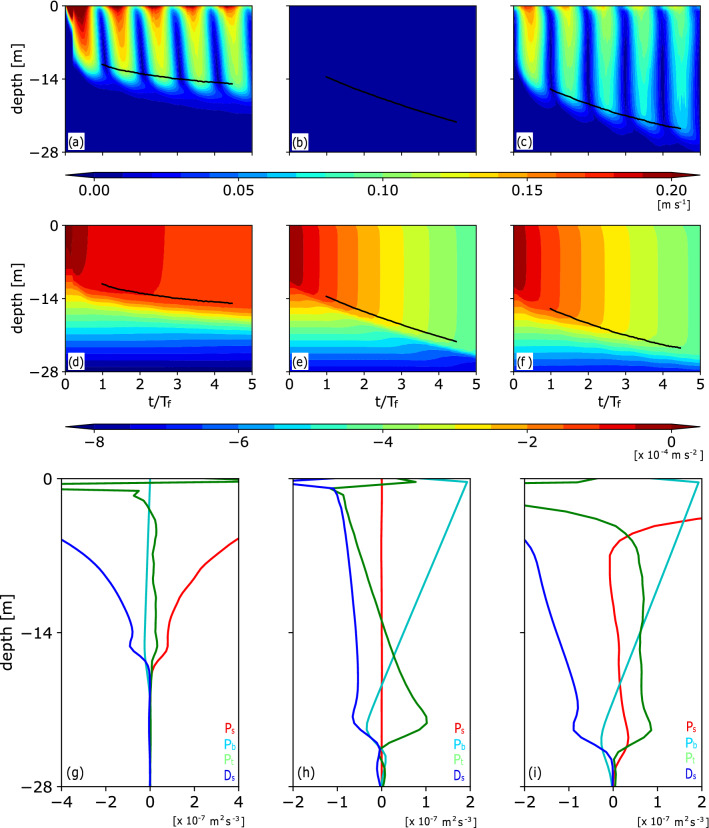
Time-depth variation of the horizontally averaged (a)–(c) current speed and (d)–(f) buoyancy (*B*) and (g)–(i) the vertical profiles of the TKE tendency terms averaged over $4.0< t/T_f < 5.0$ in the (a), (d), (g) pure ST ($U_{*}^{2} = 1.0 \times 10^{-4} \ {\mathrm{m}^{\mathrm{2}} \ \mathrm{s}^{\mathrm{-2}}}, B_f = 0$), (b), (e), (h) pure CT ($U_{*}^{2} = 0, B_f = 19.6 \times 10^{-8} \, {\mathrm{m}^2 \ \mathrm{s}^{-3}}$), and (c), (f), (i) coexisting ST and CT ($U_{*}^{2} = 1.0 \times 10^{-4} \, {\mathrm{m}^{2} \, \mathrm{s}^{-2}}, B_f = 19.6 \times 10^{-8} \, {\mathrm{m}^2 \, \mathrm{s}^{-3}}$) simulations. Initial stratification and initial MLD are $N_0 = 2.0 \times 10^{-2} \ {\mathrm{s}^{-1}}$ and $L_0 = L_D/4$, respectively. Solid lines in (a)–(f) represent the MLD.

**Figure 2:**
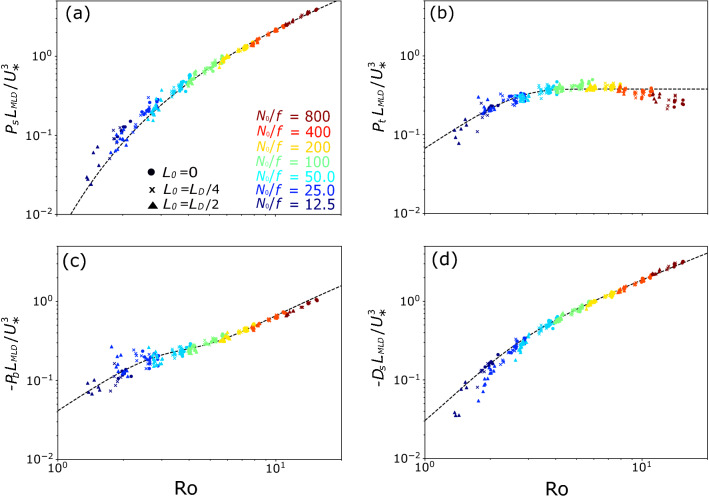
Scatter plots of $\mathrm{Ro}$ and (a) $P_s$, (b) $P_t$, (c)$-P_b$, and (d) $-D_s$ in the pure ST simulations ($B_f = 0$) averaged over $2.5< t/T_f < 3.5$ and $4.0< t/T_f < 5.0$. Each TKE tendency term was normalized by $U_*^3/L_{MLD}$. Symbols represent the initial MLD ($L_0$). Colors represent initial stratification ($N_0/f$). Dashed lines are the scalings derived in this study [Eqs. (4), (5), (7), and (8)].

**Figure 3:**
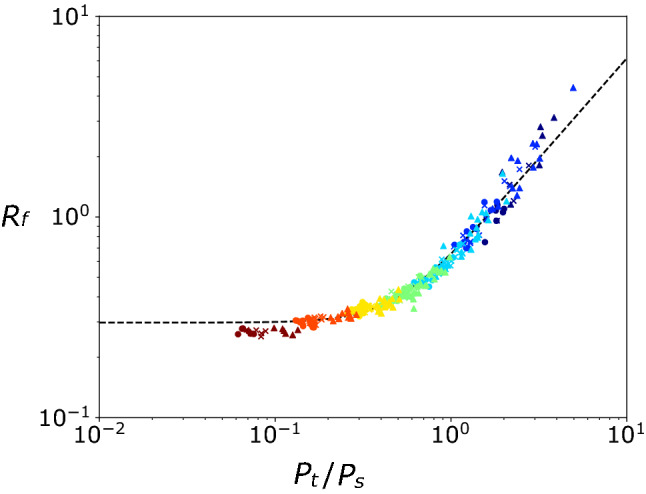
Scatter plot of $P_t/P_s$ and the flux Richardson number $R_f (\equiv -P_b/P_s)$ in the pure ST simulations ($B_f = 0$) averaged over $2.5< t/T_f < 3.5$ and $4.0< t/T_f < 5.0$. Symbols and colors are the same as in Fig. 2. Dashed line is the regression line [Eq. (6)].

Figure 1 shows the temporal depth variation of the horizontally averaged current speed and buoyancy and vertical profiles of the TKE tendency terms [see Eq. (21) in "Methods" section] averaged over $4.0<t/T_f< 5.0$, where *t* is time, and $T_f$ ($=2\pi /f$) is the inertial period. In the simulation of pure ST ($U_*^2 = 1.0 \times 10^{-4} \ {\mathrm{m}^2 \ \mathrm{s}^{-2}}$ and $B_f = 0$), the horizontal mean current speed oscillated with the inertial period (Fig. 1a). The entrainment of less buoyant water into the ML decreased the buoyancy in the ML, and the MLD ($L_{MLD}$) increased with time (Fig. 1d). Here, the MLD was defined as the depth at which the buoyancy production (${\mathcal{P}}_b$), which is a TKE tendency term [Eq. (21)], was minimum^9^. Shear production (${\mathcal{P}}_s$) was the dominant source of the pure ST, and the buoyancy production (${\mathcal{P}}_b$) and dissipation (${\mathcal{D}}_s$) were the sink terms in the ML (Fig. 1g). The convergence of the vertical transport of the TKE (${\mathcal{P}}_t$) is positive in the lower ML, but it is much smaller than ${\mathcal{P}}_s$ in this case.

In the simulation of pure CT ($U_*^2 = 0$ and $B_f = 19.6 \times 10^{-8} \ {\mathrm{m}^2 \ \mathrm{s}^{-3}}$), the MLD increased with time, although the horizontal mean current speed was zero (Fig. 1b). The buoyancy in the ML decreased because of entrainment and surface buoyancy flux (Fig. 1e). Because there was no horizontal mean velocity shear, ${\mathcal{P}}_s$ was zero (Fig. 1h). ${\mathcal{P}}_b$ was positive in the upper ML, whereas it was negative in the lower ML. ${\mathcal{P}}_t$ was negative in the upper ML and positive in the lower ML, resulting in downward transport of the TKE in the ML.

In the simulation of coexisting ST and CT ($U_*^2 = 1.0 \times 10^{-4} \ {\mathrm{m}^2 \ \mathrm{s}^{-2}}$ and $B_f = 19.6 \times 10^{-8} \ {\mathrm{m}^2 \ \mathrm{s}^{-3}}$), the current became more vertically uniform in the ML than that in the pure ST simulation (Fig. 1a and c). ${\mathcal{P}}_s$ was positive in the ML, as in the pure ST simulation (Fig. 1g and i). In the lower ML, ${\mathcal{P}}_t$ was also positive, as in the pure CT simulation, and the contribution of ${\mathcal{P}}_t$ to the TKE tendency became larger at the MLD, in contrast to that in the pure ST simulation (Fig. 1g–i). The vertical profiles of ${\mathcal{P}}_s$ and ${\mathcal{P}}_t$ in this coexisting turbulence simulation differ from the linear combinations of those in the pure turbulence simulations, indicating that ST and CT interact nonlinearly with each other, as described in previous studies^24–26^. This result implies that the entrainment buoyancy flux ($P_b$), which corresponds to the buoyancy production rate (${\mathcal{P}}_b$) at the MLD, due to coexisting ST and CT is also not represented by the linear combination of those fluxes induced by pure ST and pure CT.

### TKE tendency terms at the ML base and their scalings for pure ST and pure CT

In this subsection, we evaluate the TKE tendency terms at the ML base for pure ST and pure CT to derive scalings of the entrainment buoyancy flux for these two pure cases ($P_b^S$ and $P_b^C$, respectively). The scalings are used to quantify the nonlinear effects in coexisting ST and CT in the next subsection.

#### Pure ST case

The parameter dependence of the TKE tendency terms at the MLD in 135 pure ST simulations are reported here. Figure 2 shows a scatter plot of $\mathrm{Ro}$ ($\equiv U_*/fL_{MLD}$) and each TKE tendency term at the MLD ($P_s$, $P_t$, $-P_b$, and $-D_s$) normalized by $U_*^3/L_{MLD}$ averaged over $2.5< t/T_f < 3.5$ and $4.0< t/T_f < 5.0$. Here, $\mathrm{Ro}$ and the TKE tendency terms at the MLD ($P_s$, $P_t$, $-P_b$, and $-D_s$) were sampled every $T_f/40$ and averaged for $T_f$. All these normalized tendencies decrease with decreasing $\mathrm{Ro}$, except the normalized $P_t$ at $\mathrm{Ro}$ greater than 3, where it is almost constant. These decreases are especially rapid at $\mathrm{Ro} < 3$. Although $P_t$ is much smaller than $P_s$ in the typical case (where $\mathrm{Ro} \sim 8$) in the previous subsection, it become comparable at $\mathrm{Ro} \sim 3$ and larger at $\mathrm{Ro} \lesssim 3$.

The symbols and colors in Fig. 2 show the differences in initial MLD ($L_0$) and $N_0/f$, respectively. Note that despite the large number of simulations (135), the normalized tendency terms at the same $\mathrm{Ro}$ with different $L_0$ and $N_0/f$ collapse onto a single line. Although previous studies^35,36^ suggested that the TKE tendency terms without the Earth’s rotation depend on $\mathrm{Ri_*} \equiv \Delta B L_{MLD}/U_*^2 = N_0^2(L_{MLD}^2 - L_0^2)/2U_*^2$ (where $\Delta B$ is the vertical buoyancy difference across the MLD) and/or $\mathrm{Fr} \equiv U_*/N_0 L_{MLD}$, we found that stratification had little effect on the TKE tendency terms in the present parameter range.

By least-square fitting, we obtained the scalings of $P_s^S$ and $P_t^S$ as

4 $$\begin{aligned} P_s^S&= 0.33 \mathrm{Ro} \exp {\left( -\frac{4.2}{\mathrm{Ro}}\right) } \frac{U_*^3}{L_{MLD}}, \quad \mathrm{and} \end{aligned}$$

5 $$\begin{aligned} P_t^S&= 0.38 \tanh {\left( 0.18 \mathrm{Ro}^{1.8} \right) } \frac{U_*^3}{L_{MLD}}, \end{aligned}$$

where $P_s^S$ and $P_t^S$ are scaling-based tendencies for ST (while $P_s$, $P_t$, $-P_b$, and $-D_s$ are simulation-based tendency) and there functional forms were determined as described in "Methods" section. The scaling of $P_b$ (i.e., $P_b^S$) was derived from the relationship between $P_t/P_s$ and $P_b/P_s$. The flux Richardson number $R_f (\equiv -P_b/P_s)$ at the MLD seems to be constant if $P_s \gg P_t$^37,38^, but it is expected to change as the rotation effect increases because $P_s$ and $P_t$ became comparable at $\mathrm{Ro \lesssim 3}$ (Fig. 2a and b). From Fig. 3, the relationship between $P_t/P_s$ and $R_f$ was obtained as

6 $$\begin{aligned} R_f = \left[ 0.30^{\frac{5}{2}} + \left( 0.62 \frac{P_t}{P_s}\right) ^{\frac{5}{2}} \right] ^{\frac{2}{5}}; \end{aligned}$$

that is,

7 $$\begin{aligned} P_b^S = - \left[ \left( 0.30 P_s^S \right) ^{\frac{5}{2}} + \left( 0.62 P_t^S\right) ^{\frac{5}{2}}\right] ^{\frac{2}{5}}. \end{aligned}$$

Finally, the scaling of $D_s$ (i.e., $D_s^S$) was obtained as

8 $$\begin{aligned} D_s^S = - (P_s^S + P_t^S + P_b^S), \end{aligned}$$

where we assumed almost steady TKE at the MLD (due to slow deepening of the ML). These relations reproduce well the simulated dependence of the TKE tendency terms on $\mathrm{Ro}$ (Fig. 2).

**Figure 4:**
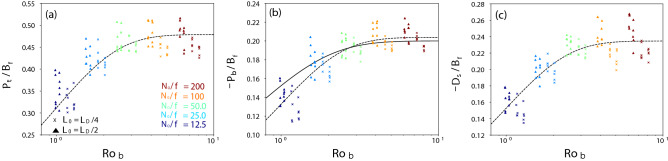
Scatter plots of ${\mathrm{Ro}_{\mathrm{b}}}$ and (a) $P_t$, (b)$-P_b$, and (c) $-D_s$ in the pure CT simulations ($U_*^2 = 0$) averaged over $2.5< t/T_f < 3.5$ and $4.0< t/T_f < 5.0$. Each TKE tendency term was normalized by $B_f$. Symbols and colors are the same as in Fig. 2. Dashed lines are the scalings derived in this study [Eqs. (9)–(11)], and solid line in (b) is the scaling of^19^ [$0.2 \tanh ({\mathrm{Ro}_{\mathrm{b}}}^{0.69})$].

**Figure 5:**
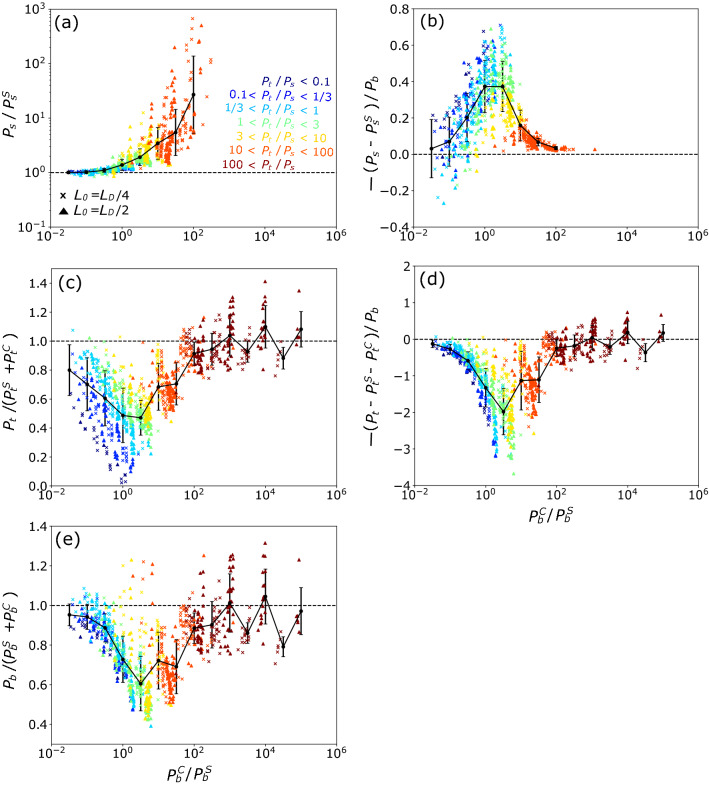
Scatter plots of (a) $P_b^C/P_b^S$ and $P_s/P_s^S$, (b) $P_b^C/P_b^S$ and $(P_s-P_s^S)/P_b$, (c) $P_b^C/P_b^S$ and $P_t/(P_t^S+P_t^C)$, (d) $P_b^C/P_b^S$ and $[P_t-(P_t^S+P_t^W)]/P_b$, and (e) $P_b^C/P_b^S$ and $P_b/(P_b^S+P_b^C)$ in the coexisting ST and CT simulations. Symbols are the same as in Fig. 2. Colors represent $P_t/P_s$. Black circle and bar show the averaged value and standard deviation, respectively.

**Figure 6:**
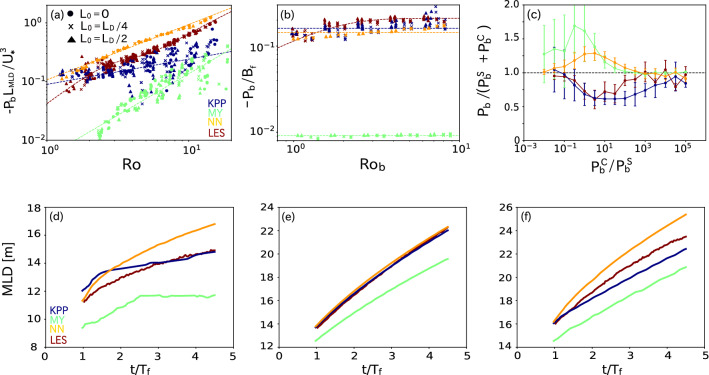
Same as (a) Fig. 2c, (b) Fig. 4b, and (c) Fig. 5e but for the results of the LES (red) and 1D simulations with the KPP (blue), MY (green), and NN (orange) schemes. Temporal variation of the MLD in the simulations with the typical parameters of (d) pure ST, (e) pure CT, and (f) coexisting ST and CT. The parameters used in (d)–(f) are the same as those in Fig. 1a–c, respectively. Time is normalized by $T_f$. Colors represent the LES (red) and the KPP (blue), MY (green), and NN (orange) schemes.

#### Pure CT case

For pure CT, the TKE tendencies are suggested to depend on $B_f$ and ${\mathrm{Ro}_{\mathrm{b}}} \equiv W_*/fL_{MLD}$^18–20^. Figure 4 shows a scatter plot of ${\mathrm{Ro}_{\mathrm{b}}}$ and each TKE tendency term (except $P_s$, because $P_s=0$ in the pure CT cases) normalized by $B_f$ in 50 pure CT simulations. At ${\mathrm{Ro}_{\mathrm{b}}} >3$, all the normalized tendency terms are almost constant, and $P_b^C \cong - 0.2 B_f$, which is consistent with previous studies^9,19^. At ${\mathrm{Ro}_{\mathrm{b}}} <3$, they decrease with decreasing ${\mathrm{Ro}_{\mathrm{b}}}$. Because the scaling of Wang^19^ [$P_b^C = - 0.2 \tanh ({{\mathrm{Ro}_{\mathrm{b}}}^{0.69}}) B_f$] slightly overestimated $P_b$ at ${\mathrm{Ro}_{\mathrm{b}}} \sim 1$ (Fig. 4b), the scaling was modified in this study. By least-square fitting, we obtained

9 $$\begin{aligned} P_t^C&= 0.48 \tanh {\left( 0.78 {\mathrm{Ro}_{\mathrm{b}}}^{0.83} \right) } B_f, \end{aligned}$$

10 $$\begin{aligned} P_b^C&= - 0.20 \tanh {\left( 0.78 {\mathrm{Ro}_{\mathrm{b}}}^{0.83} \right) } B_f, \quad \mathrm{and} \end{aligned}$$

11 $$\begin{aligned} D_s^C&= - 0.23 \tanh {\left( 0.78 {\mathrm{Ro}_{\mathrm{b}}}^{0.83} \right) } B_f, \end{aligned}$$

where $P_t^C$, $P_b^C$, and $D_s^C$ are scaling-based tendencies for CT. Here, $P_t^C + P_b^C + D_s^C \ne 0$ because the temporal change in the TKE is large (i.e., ongoing ML deepening occurs), in contrast to that in the pure ST cases.

### Nonlinear effects of ST and CT interaction at the ML base

Using the scalings of the TKE tendency terms for pure ST ($P_s^S$ [Eq. (4)], $P_t^S$ [Eq. (5)], and $P_b^S$ [Eq. (7)]) and pure CT ($P_t^C$ [Eq. (9)] and $P_b^C$ [Eq. (10)]) and simulation-based tendency of $P_s$, $P_t$ and $P_b$ for 450 simulatinos of coexisting ST and CT, the nonlinear effects of ST and CT interaction at the MLD are evaluated. Figure 5a shows $P_s/P_s^S$ (ratio of the simulated shear production with CT to the scaling-based shear production due to pure ST) as a function of $P_b^C/P_b^S$ (a measure of CT relative to ST). Note that $P_s/P_s^S$ was excluded if $P_t/P_s > 100$ because $P_s$ has little effect on TKE tendency. $P_s/P_s^S$ increases with $P_b^C/P_b^S$, indicating that ST is intensified by CT. For example, $P_s$ becomes 20–30 times larger than $P_s^S$ at $P_b^C/P_b^S \cong 10^2$. Note, however, that as CT becomes more dominant, $P_t$ has more impact on the TKE tendency than $P_s$ [see the color representing the intensity of $P_t$ relative to $P_s$ ($P_t/P_s$) in Fig. 5a]. Figure 5b shows $-(P_s-P_s^S)/P_b$, a ratio of the increased shear production by CT ($P_s-P_s^S$) to the entrainment buoyancy flux ($P_b$), as a function of $P_b^C/P_b^S$. The impact of the increased shear by CT on the entrainment is found largest at around $P_b^C/P_b^S = 10^{1/2}$.

Previous studies^14,39^, on the other hand, suggested that CT is inhibited by ST. To see this effect, we plot $P_t/(P_t^S+P_t^C)$ as a function of $P_b^C/P_b^S$ in Fig. 5c. Here, $P_t$ is a measure of CT at the MLD, and the ratio of $P_t$ to $P_t^S+P_t^C$ represents the intensity of the nonlinear interaction effects. $P_t$ was $40 \ \%$ smaller than $P_t^S + P_t^C$ at $10^{-1/2}< P_b^C/P_b^S < 10^{1/2}$, indicating that ST and CT interact nonlinearly to decrease $P_t$ in this parameter range. This indicates that ST disrupts coherent structure of pressure and/or TKE and vertical velocity [see Eq. (21) in "Methods" section] associated with convective motion (CT). This $P_t$ decrease [$P_t-\left( P_t^S+P_t^C\right)$] by the interaction contributes more to $P_b$ than the increased $P_s$ by the interaction (Fig. 5b and d). Consequently, $P_b$ became $30 \ \%$ smaller than $P_b^S + P_b^C$ at $1< P_b^C/P_b^S < 10^{3/2}$ (Fig. 5e).

### Entrainment flux in the ocean mixing parameterization schemes used in OGCMs

In the previous subsection, we suggested that the nonlinear interaction between ST and CT likely inhibits the entrainment at the ML base at $1< P_b^C/P_b^S < 10^{3/2}$. To accurately simulate the ML-related processes using OGCMs, the mixing parameterization schemes should reproduce the entrainment buoyancy flux of the nonlinear interaction effects as well as those of the pure turbulences. To see the extent to which the schemes reproduce the entrainment flux of the pure turbulences and the nonlinear effects, one-dimensional (1D) simulations with the mixing parameterization schemes were performed, and the results are compared to the LES results in this subsection.

The mixing parameterization schemes tested in this study were the KPP scheme^6^, level 2.5 MY scheme^5,40,41^, and level 2.5 Nakanishi–Niino (NN) scheme^42–44^. The NN scheme is a modified version of the MY scheme and is used in ocean models^45^ as well as atmospheric models^46,47^. The boundary and initial conditions were the same as those in the LES. The detail configuration for 1D simulations is described in "Methods" section.

Figure 6 shows scatter plots of $\mathrm{Ro}$ ($\equiv U_*/fL_{MLD}$) and $P_b^S$ normalized by $U_*^3/L_{MLD}$ for the 1D pure ST simulations (cf. Fig. 2c), ${\mathrm{Ro}_{\mathrm{b}}} (\equiv W_*/fL_{MLD})$ and $P_b^C$ normalized by $B_f$ for the 1D pure CT simulations (cf. Fig. 4c), and $P_b^C/P_b^S$ and $P_b$ normalized by $P_b^S + P_b^C$ in the 1D simulations of coexisting ST and CT (cf. Fig. 5c). Figure 6 also shows the temporal change in MLD in the 1D simulations using the typical parameters used in the LESs shown in Fig. 1. For pure ST, the normalized $P_b^S$s in the KPP and MY schemes show more scattering than those in the LES at $\mathrm{Ro} > 3$, suggesting that these schemes are likely affected by other factors such as $\mathrm{Ri_*}$ and/or $\mathrm{Fr}$, which had little effect on $P_b^C$ in the LESs in the present parameter range (Fig. 6a). Because these normalized $P_b^s$s were also underestimated, the ML deepened less from $t/T_f=1$ to $t/T_f=4.5$ than it did in the LES (Fig. 6d). The NN scheme successfully reproduced the dependence of $P_b^S$ on Ro with less scatter, but it overestimated $P_b^S$ (Fig. 6a) and thus the MLD (Fig. 6d).

The scalings of $P_b^S$ in these schemes were evaluated for later use. For simplicity, $P_b^S$ was assumed to be proportional to $\mathrm{Ro}^d$, where *d* is constant. By least-square fitting, we obtained

12 $$\begin{aligned} P_b^{S-KPP}&= - 0.090 \mathrm{Ro}^{0.43} \frac{U_*^3}{L_{MLD}}, \end{aligned}$$

13 $$\begin{aligned} P_b^{S-MY}&= - 0.0052 \mathrm{Ro}^{1.4} \frac{U_*^3}{L_{MLD}}, \quad \mathrm{and} \end{aligned}$$

14 $$\begin{aligned} P_b^{S-NN}&= - 0.11 \mathrm{Ro}^{0.94} \frac{U_*^3}{L_{MLD}}. \end{aligned}$$

For pure CT, on the other hand, the normalized $P_b^C$s in the KPP and NN schemes were similar to those of the LES, whereas those in the MY scheme were much smaller (Fig. 6b). As a result, the MLDs were well reproduced by the KPP and NN schemes and underestimated by the MY scheme (Fig. 6e). The decreases in $P_b^C$ with decreasing ${\mathrm{Ro}_{\mathrm{b}}}$ were smaller in all of these schemes than in the LES (Fig. 6b), probably because they do not include the effects of Earth’s rotation. Here, we assume $P_b^C \propto B_f$ and derive the scalings of $P_b^C$ in these schemes by least-square fitting as

15 $$\begin{aligned} P_b^{C-KPP}&= - 0.16 B_f, \end{aligned}$$

16 $$\begin{aligned} P_b^{C-MY}&= - 0.0091 B_f, \quad \mathrm{and} \end{aligned}$$

17 $$\begin{aligned} P_b^{C-NN}&= - 0.14 B_f. \end{aligned}$$

The nonlinear interaction effects between ST and CT in these mixing parameterization schemes were quantified using these scalings [Eqs. (12)–(17)] (Fig. 6c). The $P_b$s in the MY and NN schemes are greater than $P_b^S+P_b^C$, indicating that they tend to represent the interaction effects in an opposite sense. The MLD was underestimated by the MY scheme (Fig. 6f) owing to the underestimations of $P_b^S$ (Fig. 6a) and $P_b^C$ (Fig. 6b), despite the failure to reproduce the nonlinear effects (Fig. 6c). On the other hand, the MLD was overestimated by the NN scheme (Fig. 6f) because of the overestimation of $P_b^S$ (Fig. 6a) and the failure to reproduce the nonlinear effects (Fig. 6c). The KPP scheme successfully reproduced the nonlinear effects except at $P_b^C/P_b^S > 10$ (Fig. 6c), although the underestimation of $P_b^S$ resulted in the underestimation of the MLD (Fig. 6f). Note that the above differences between the LES and mixing schemes would become smaller by tuning empirical parameters in the schemes, though it seems uneasy to reduce the differences in the pure and coexisting turbulence regimes simultaneously.

**Figure 7:**
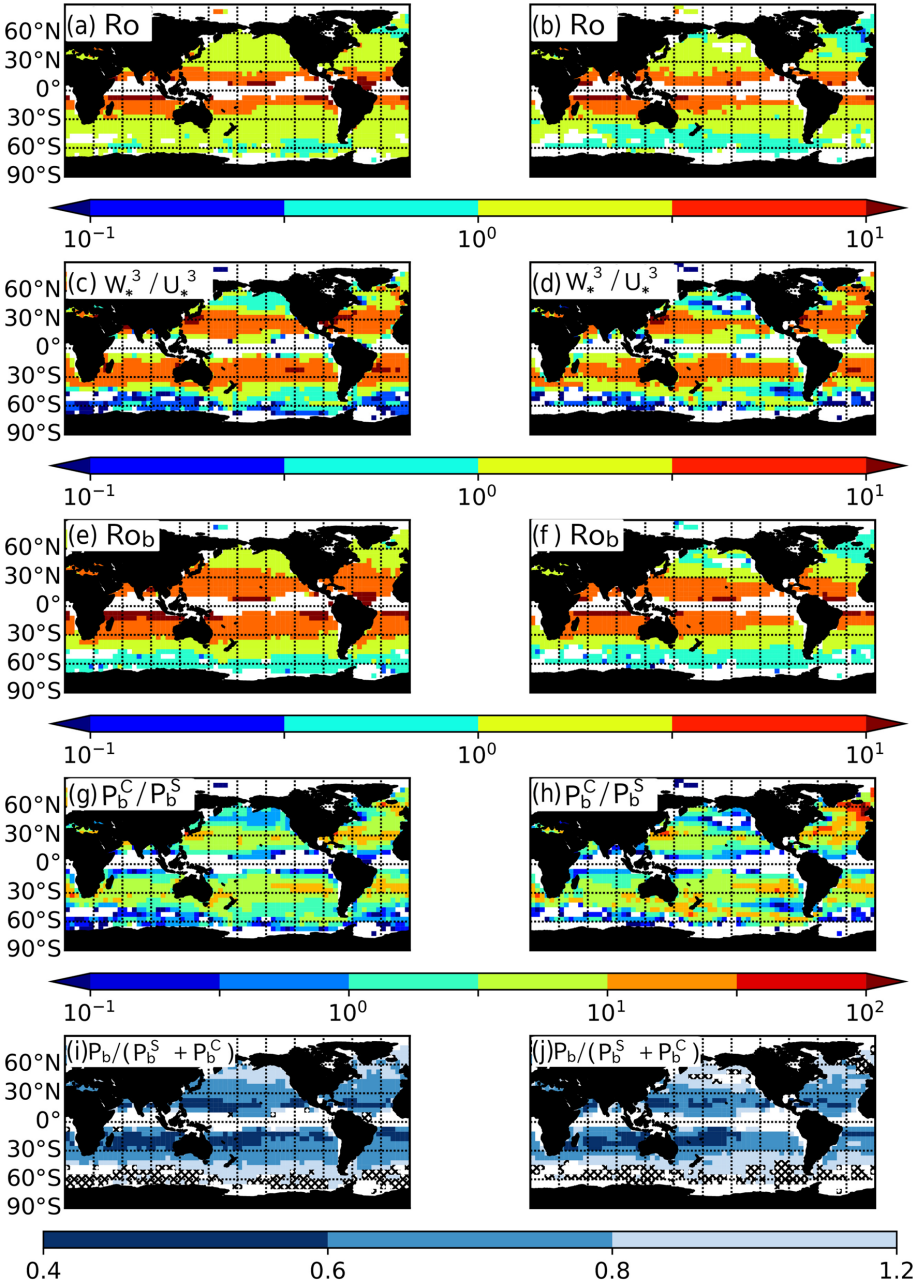
Seasonal (three month) averages of (a), (b) $\mathrm{Ro} (\equiv U_*/fL_{MLD})$, (c), (d) $W_*^3/U_*^3$, (e), (f) ${\mathrm{Ro}_{\mathrm{b}}} (\equiv W_*/fL_{MLD})$, (g), (h) $P_b^C/P_b^S$, and (i), (j) $P_b/(P_b^S+P_b^C)$ in (a), (c), (e), (g), (i) autumn and (b), (d), (f), (h), (j) winter estimated from observations in 2001–2010. $P_b/(P_b^S+P_b^C)$ in (i) and (j) were evaluated from the observed $(\mathrm{Ro}, W_*^3/U_*^3)$ and the simulated bin-averaged relationship between $(\mathrm{Ro}, W_*^3/U_*^3)$, and $P_b/(P_b^S+P_b^C)$ shown in Fig. 8b.

**Figure 8:**
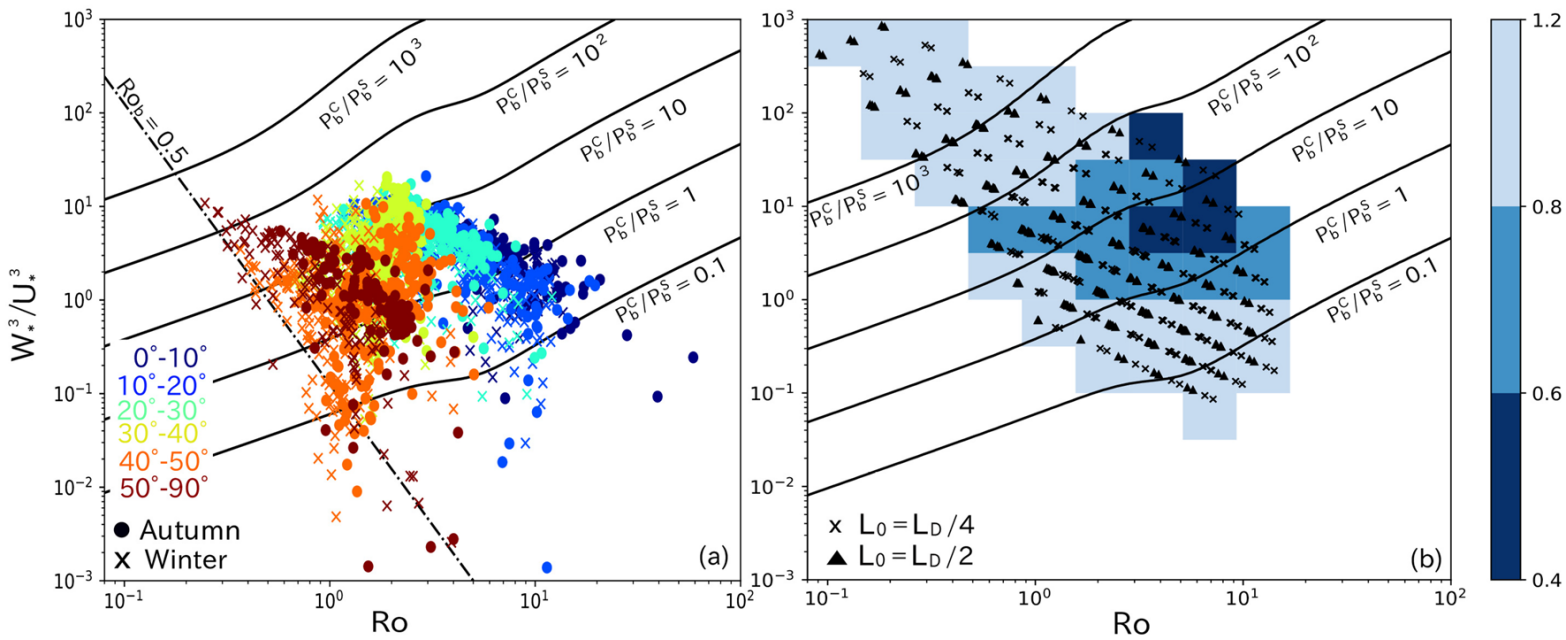
Scatter plots of $\mathrm{Ro}$ and $W_*^3/U_*^3$ calculated from (a) observed data and (b) LES data. Symbols in (a) and (b) represent seasons and initial MLD ($L_0$), respectively. Color represents latitude in (a) and bin-averaged $P_b/(P_b^S+P_b^C)$ in (b). Solid lines are contour lines of $P_b^C/P_b^S$, and dash-dotted line in (a) is contour line of ${\mathrm{Ro}_{\mathrm{b}}} = 0.5$.

## Discussion

In this section, we estimate the global distribution of the intensity of the nonlinear effects in the real ocean. The parameters $\mathrm{Ro}$, $W_*^3/U_*^3$, and ${\mathrm{Ro}_{\mathrm{b}}}$ were calculated from the $U_*$, $B_f$, and $L_{MLD}$ in observed and reanalysis data in autumn and winter (see "Methods" section) and are shown in Fig. 7. (Note that the $U_*^3/L_{MLD}$-normalized $P_b^S$ and $P_b^C$ depend only on these parameters.) Here, data at $B_f < 0$ were excluded from the analysis. (These data were often found near the equator.) Figures 7a and b show that $\mathrm{Ro}$ is larger due to smaller *f* and amounts to $10^{1/2} (= 3.2)$ or more in tropical regions ($< 20^\circ$). On the other hand, $W_*^3/U_*^3$ is small ($< 10^{1/2}$) at lower latitude than $15^\circ$ and higher latitude than $40^\circ$ and large ($> 10^{1/2}$) at mid-latitude ($15^\circ$–$40^\circ$) (Fig. 7c and d). As a result, ST is more dominant than CT ($P_b^C/P_b^S < 1$) at the low and high latitudes (Fig. 7g and h). At higher latitude than $45^\circ \ \mathrm{N}$ in the North Atlantic, $W_*^3/U_*^3$ is also large (Fig. 7c and d), and hence $P_b^C/P_b^W > 10^{3/2}$ (Fig. 7g and h), especially in winter. However, ${\mathrm{Ro}_{\mathrm{b}}} < 1$ in winter (Fig. 7e and f) suggests that deep water formation in the North Atlantic is inhibited by Earth’s rotation^17^. Note that at mid-latitudes, $P_b^C/P_b^S$ ranges from 1 to $10^{3/2}$, indicating that the nonlinear interaction between ST and CT is expected.

To estimate geological distribution of the expected nonlinear interaction intensity (Fig. 7i and j), we used Fig. 8, where scatter plots of $\mathrm{Ro}$ and $W_*^3/U_*^3$ from the observed (Fig. 8a) and simulated (Fig. 8b) data are shown. In Fig. 8b, the intensity of the nonlinear effects [$P_b/(P_b^S+P_b^C)$] averaged over bins on $(\mathrm{Ro}, W_*^3/U_*^3)$ space is also shown. Here, the observed $(\mathrm{Ro}, W_*^3/U_*^3)$ at a certain grid point (Fig. 7a-d and 8a) was converted to $P_b/(P_b^S+P_b^C)$ using simulated relation between $(\mathrm{Ro}, W_*^3/U_*^3)$ and bin-averaged $P_b/(P_b^S+P_b^C)$ in Fig. 8b, and this was considered as observed $P_b/(P_b^S+P_b^C)$. The cross-hatching in Fig. 7i and j represents the region where $\mathrm{Ro}$ and $W_*^3/U_*^3$ are outside of the simulated parameter range. These figures show that our simulations covered most of the observed pairs of $\mathrm{Ro}$ and $W_*^3/U_*^3$, except in the Southern Ocean, where surface cooling is weak relative to wind stress ($W_*^3/U_*^3 < 0.1$) and ST is expected to strongly dominate CT. The observed $P_b/(P_b^S+P_b^C)$ is less than 0.8 at mid-latitudes and 0.6 at some region between $15^\circ$ and $25^\circ$, indicating that the nonlinear interaction between ST and CT probably suppresses the entrainment at the ML base there. Because some mixing parameterization schemes such as the MY and NN schemes cannot reproduce the nonlinear effects, the fact that the schemes did not successfully reproduce the effect of nonlinear interaction between ST and CT might explain the mid-latitude deep MLD biases in winter observed in some OGCMs as seen in Fig. 1 of Belcher et al.^28^ and Fig. 11 of Tsujino et al.^34^. [More than half OGCMs in the Coupled Model Intercomparison Project phase 6 evaluated by Tsujino et al.^34^ adopted schemes similar to the MY and NN schemes (1.5 or higher order turbulence closure schemes), though each of the schemes used slightly different parameterizations and/or tuning parameters from those of the MY and NN schemes.] This result suggests that mixing parameterization schemes need to be checked and improved (if necessary) to correctly represent ocean surface mixing due to ST and CT.

In this study, we found the nonlinear interaction between ST and CT is expected large in mid-latitude ML in the ocean. However, the interaction mechanism remains to be investigated in more detail. We also found that the KPP, MY, and NN schemes do not well represent pure ST mixing. Because ST likely plays more role in the ocean than in the atmosphere, we consider that this issue should not be overlooked in ML mixing schemes. Effects of wave and/or time-variying forcing as well as heterogeneous background environment (such as ocean front) also need to be considered simultaneously for realistic ML simulation in the OGCMs. These will be studied in future.

## Methods

### Simulations and data

#### Numerical model and experimental configurations for large-eddy simulations

The LES model used in this study is the same as that used in Ushijima and Yoshikawa^48,49^. The governing equations are the momentum equation, continuity equation, and advection–diffusion equation of buoyancy under the incompressible, *f*-plane, Boussinesq, and rigid-lid approximations. Subgrid-scale parameterization follows the method described by Deardorff^50^ and Maronga et al.^51^. At the surface, constant wind stress ($\rho _0 U_*^2$, where $\rho _0 = 1.0 \times 10^{3} \ \mathrm{kg \ m^{-3}}$ is the reference water density) and buoyancy flux ($B_f$) were imposed. We also imposed subgrid-scale shear production at the surface. At the bottom, the free-slip condition and no-buoyancy flux condition were imposed. The lateral boundaries were periodic in both directions. The initial stratification ($N \equiv \sqrt{\partial B/\partial z}$, where *B* is the horizontally averaged buoyancy) was zero ($N = 0$) from the surface ($z = 0$) to the initial MLD ($z = - L_0$) and constant ($N = N_0$) from $z = - L_0$ to the bottom of the ocean ($z=-L_D$, where $L_D$ is the domain length).

To evaluate the buoyancy entrainment flux due to pure ST, pure CT, and coexisting ST and CT, simulations were performed with several values of the momentum flux ($U_*^2$), surface buoyancy flux ($B_f$), initial stratification ($N_{0} = 0.125, 0.25, 0.5, 1.0$, and $2.0 \times 10^{-2} \ \mathrm{s^{-1} }$, corresponding to temperature changes of 0.078, 0.31, 1.3, 5, and $20 \ \mathrm{K}$ in $100 \ \mathrm{m}$), initial MLD ($L_0$), and Coriolis parameter (*f*). In the simulations of pure ST, we set $U_*^2 = 0.5, 1.0$, and $2.0 \times 10^{-4} \ \mathrm{m^2 \ s^{-2}}$ (corresponding to wind speeds at 10 m height of 7, 10, and $14 \ \mathrm{m \ s^{-1}}$), $B_f = 0$, $L_0 = 0, L_D/4$, and $L_D/2$, and $f = 2.5, 5.0$, and $10 \times 10^{-5} \ \mathrm{s^{-1}}$ (corresponding to latitudes of $10^{\circ }, 20^{\circ }$, and $40^{\circ } \ \mathrm{N}$). In the simulations of pure CT, we set $U_*^2 = 0$, $B_f = 1.225, 2.45, 4.9, 9.8$, and $19.6 \times 10^{-8} \ \mathrm{m^2 \ s^{-3}}$ (corresponding to surface cooling of 25, 50, 100, 200, and $400 \ \mathrm{W \ m^{-2}}$), $L_0 = L_D/4$ and $L_D/2$, and $f = 10 \times 10^{-5} \ \mathrm{s^{-1}}$. A total of 135 and 50 simulations were performed for pure ST and pure CT, respectively. To examine the nonlinear interaction between ST and CT, a total of 450 simulations with $U_*^2 = 0.5, 1.0$, and $2.0 \times 10^{-4} \ \mathrm{m^2 \ s^{-2}}$, $B_f = 1.225, 2.45, 4.9, 9.8$, and $19.6 \times 10^{-8} \ \mathrm{m^2 \ s^{-3}}$, $L_0 = L_D/4$ and $L_D/2$, and $f = 2.5, 5.0$, and $10 \times 10^{-5} \ \mathrm{s^{-1}}$ were performed. These parameters are determined from the typical values of the observed climatologies in autumn and winter.

The governing equations were discretized using the second-order finite-difference scheme and integrated in time using the second-order Runge–Kutta scheme. The number of grid cells was $64 \times 64 \times 64$, and the grid spacing was uniform. The domain size, $L_D \times L_D \times L_D$, was varied according to the friction velocity ($U_*$), buoyancy flux ($B_f$), initial stratification ($N_0$), and Coriolis parameter (*f*). In the simulations of pure ST and coexisting ST and CT, $L_D$ was set to $4 L_{P73} (1 + 5.1 B_f/U_*^2N_0)$, where $L_{P73} \equiv U_*/\sqrt{N_0f}$ is the MLD scale characterizing the wind-induced ML in the stratified ocean under the Earth’s rotation^49,52^, whereas $L_D$ was set to $17 \sqrt{B_f/N^2f}$ in the simulations of pure CT. We performed several LESs with quarter-grid spacing but the same domain size and found that the TKE tendency terms obtained in the simulations with higher resolution were almost the same as those obtained with the original resolution. The dependence on the resolution is discussed in detail in Supplementary Information. The integration was continued for $5T_f$, where $T_f = 2\pi /f$ is the inertial period.

#### Experimental configurations for one-dimensional simulations with mixing parameterization schemes

The governing equations for one-dimensional (1D) simulations are momentum equation and diffusive equation of buoyancy,

18 $$\begin{aligned} \frac{\partial U}{\partial t} - fV&= \frac{\partial }{\partial z}\left( K_M \frac{\partial U}{\partial z} \right) , \end{aligned}$$

19 $$\begin{aligned} \frac{\partial V}{\partial t} + fU&= \frac{\partial }{\partial z}\left( K_M \frac{\partial V}{\partial z} \right) , \quad \mathrm{and} \end{aligned}$$

20 $$\begin{aligned} \frac{\partial B}{\partial t}&= \frac{\partial }{\partial z}\left( K_S \frac{\partial U}{\partial z} \right) , \end{aligned}$$

where (*U*, *V*) is the horizontal velocity components, *B* is buoyancy, and $K_M$ and $K_S$ are the eddy viscosity and diffusivity, determined in the KPP^6^, MY^5,40,41^, or NN^42–44^ schemes, respectively. The boundary condition at the surface and the bottom, domain depth ($L_D$), and the number of the vertical grid cells, are same as those in the LES. A total of 135, 50, and 450 simulations for pure ST, pure CT, and coexisting ST and CT were respectively performed for each 1D experiment with different mixing schemes with same momentum flux ($U_*$), buoyancy flux ($B_f$), initial stratification ($N_0$), initial MLD ($L_0$), and Colioris parameter as those in the LES.

#### Observed and reanalysis data

Data were analyzed for autumn (October, November, and December in the northern hemisphere and April, May, and June in the southern hemisphere) and winter (January, February, and March in the northern hemisphere and July, August, and September in the southern hemisphere), when ST and CT are typically expected to coexist. The climatology of the ML temperature ($T_{ML}$) and salinity ($S_{ML}$) as well as the MLD ($L_{MLD}$) of the mixed layer Argo dataset, gridpoint value (MILA-GPV)^53^ were used. The surface fluxes were the 6-hourly momentum fluxes ($\tau _x, \tau _y$), net heat flux ($H_f$), and freshwater flux ($E-P$) from the National Centers for Environmental Prediction (NCEP) data^54^ for 2001–2010, where *E* and *P* are the evaporation rate and precipitation rate, respectively. The shortwave radiation was assumed not to penetrate below the surface for simplicity, and the evaporation rate was estimated from the latent heat flux with the latent heat vaporization of water^55^. These fluxes were converted to the friction velocity $U_* [= ({\tau _x^2+\tau _y^2})^{1/4}/\rho _0^{1/2}]$ and buoyancy flux $B_f [= - \alpha g H_f/\rho _0 C_a + \beta g (E-P) S_{ML}]$. Here, $\rho _0 (=1.0 \times 10^3 \ \mathrm{kg \ m^{-3}})$ and $C_a (=4.0 \times 10^3 \ \mathrm{J \ kg^{-1} \ C^{-1}})$ are the reference density and heat capacity of water, respectively. The thermal expansion rate ($\alpha$) and haline contraction rate ($\beta$) were calculated from $T_{ML}$ and $S_{ML}$ using the equation of state for seawater^56^. The momentum flux, buoyancy flux, and MLD were averaged into seasonal (three-month) climatological data points. The horizontal resolution of the data was $5^\circ \times 5^\circ$.

### Analysis

#### TKE tendency terms in the LES

The TKE tendency terms were calculated as

21 $$\begin{aligned} \frac{\partial }{\partial t}\left( \frac{1}{2} \overline{u_{k}^{\prime } u_{k}^{\prime }} + {\overline{e}} \right) = \underbrace{- (\overline{u_k^\prime w^\prime } - 2\overline{\nu s_{k3}}) \frac{\partial \overline{u_k}}{\partial z}}_{{\mathcal{P}}_s} \underbrace{+ \overline{w^\prime b^\prime } - \overline{\kappa \frac{\partial b}{\partial z}}}_{{\mathcal{P}}_b} \underbrace{- \frac{\partial }{\partial z}\left( \frac{1}{2}\overline{u_k^\prime u_k^\prime w^\prime } + \frac{\overline{\pi ^\prime w^\prime }}{\rho _0} + \overline{w^\prime e} + 2 \overline{u_k^{\prime } \nu s_{k3}} + 2 \overline{\nu \frac{\partial e}{\partial z}} \right) }_{{\mathcal{P}}_t} \underbrace{ - {\overline{\varepsilon }}}_{{\mathcal{D}}_s}, \end{aligned}$$

where $u_i$ represents the velocity components $(u,\upsilon ,w)$ in the $x_i$ direction, $x_i (i = 1, 2, 3)$ denotes the Cartesian coordinates (*x*, *y*, *z*), *b* is buoyancy, $\pi = p +2 \rho _0 e/3$ is modified pressure, *p* is pressure, and $s_{ij} \equiv (\partial u_i/\partial x_j + \partial u_j/\partial x_i)/2$. The subgrid-scale kinetic energy (*e*), eddy viscosity ($\nu$), eddy diffusivity ($\kappa$), and dissipation rate ($\varepsilon$) were calculated using sub-grid scale parameterization^50,51^. The overbar represents the horizontal average, and the prime indicates anomalies from the horizontal average. In the above equation, ${\mathcal{P}}_s$, ${\mathcal{P}}_b$, ${\mathcal{P}}_t$, and ${\mathcal{D}}_s$ represent the rates of shear production, buoyancy production, convergence of vertical transport of the TKE, and dissipation of the TKE, respectively. (Note that they are a function of *z*.)

#### Functional forms of the $P_s^S$ and $P_t^S$ scalings

In the pure ST simulations, the normalized $P_s$ is almost linearly proportional to $\mathrm{Ro}$ at large $\mathrm{Ro}$, but the slope increases for smaller $\mathrm{Ro}$ (Fig. 2a). Under neutral stratification, the vertical shear of the horizontal velocity (the Ekman velocity shear) decreases with depth (|*z*|) as $\exp ({-|z |/L_{EKD}})$, where $L_{EKD} \equiv U_*/f$ is the depth of the turbulent Ekman layer^21^. Therefore, the vertical shear at the MLD is expected to be proportional to $\exp [-c L_{MLD}/(U_*/f)] = \exp (-c/\mathrm{Ro})$, where *c* is a constant. Consequently, we assume that the normalized $P_s$ is proportional to $\mathrm{Ro}\exp ({c/\mathrm{Ro}})$.

On the other hand, the normalized $P_t$ at $\mathrm{Ro} < 3$ decreases with decreasing $\mathrm{Ro}$ but does not vary significantly with $\mathrm{Ro}$ at $\mathrm{Ro} > 3$ (Fig. 2b). Because the variation of this normalized $P_t^S$ with $\mathrm{Ro}$ is similar to that of the normalized $P_t^C$ with ${\mathrm{Ro}_{\mathrm{b}}}$ in the pure CT simulations, we assume that the normalized $P_t^S$ has the same functional form as the normalized $P_t^C$ [Eq. (9)].

## Supplementary Information

Supplementary Information 1.

Supplementary Information 2.

## Data Availability

The simulated data are available at https://fsv.iimc.kyoto-u.ac.jp/public/dkIIAARcbEnApIoBKkt-lhozyjprHhuan8hCdrVpdDkw. The data used for MILA-GPV and NCEP reanalysis were downloaded from https://www.jamstec.go.jp/ARGO/argo_web/MILAGPV/index.html and https://psl.noaa.gov/data/gridded/data.ncep.reanalysis.html, respectively.
