# Supplementary material for: Nonlinearly interacting entrainment due to shear and convection in the surface ocean
Source: Sci Rep. 2022 Jun 14;12:9899. doi: 10.1038/s41598-022-14098-w (PMC9198105; doi:10.1038/s41598-022-14098-w)
Supplement: Supplementary file 1 — Supplementary Information 1. [file 41598_2022_14098_MOESM1_ESM.doc]

Title: Supplementary Discussion

This Supplementary Discussion describes the dependence on the resolution of our simulations and includes one related figure to the discussion.
