# Supplementary material for: Nonlinearly interacting entrainment due to shear and convection in the surface ocean
Source: Sci Rep. 2022 Jun 14;12:9899. doi: 10.1038/s41598-022-14098-w (PMC9198105; doi:10.1038/s41598-022-14098-w)
Supplement: Supplementary file 2 — Supplementary Information 2. [file 41598_2022_14098_MOESM2_ESM.pdf]

Supplementary Discussion for "Nonlinearly interacting entrainment due to shear and convection in the surface ocean"

Yusuke Ushijima[1,2,*] **and Yutaka Yoshikawa**[3]

[1]Research Promotion Department, Japan Meteorological Business Support Center, Tsukuba 305-0052, Japan

[2]Department of Atmosphere, Ocean, and Earth System Modeling Research, Meteorological Research Institute, Tsukuba 305-0052, Japan

[3]Division of Earth and Planetary Sciences, Graduate School of Science, Kyoto University, Kyoto 606-8502, Japan

[*]usijimay@mri-jma.go.jp

Contents of this file

1. Supplementary Discussion: Dependence of entrainment buoyancy flux on resolution of LESs

2. Figure S1

Supplementary Discussion

This Supplementary Discussion describes the dependence on the resolution of our simulations and includes one related figure to the discussion.

Supplementary Discussion: Dependence of entrainment buoyancy flux on resolution of LESs

The dependence of the entrainment buoyancy flux on the resolution of the large-eddy simulations (LESs) is discussed here. To this aim, several LESs with quarter-grid spacing but the same domain size to the original simulations described in Methods section were additionally performed. (The number of grid cells of these simulations was $256 \times 256 \times 256$.) In the simulations with higher resolution, we set $U_*^2 = 1.0 \times 10^{-4}$ m^2 s^{-2}, $B_f = 0$, $N_0 = 0.125, 0.5$, and 2.0×10^{-2} s^{-1}, $L_0 = L_D/2$, and $f = 2.5$ and 10×10^{-5} s^{-1} for pure ST, $U_*^2 = 0$, $B_f = 1.225, 4.9$, and 19.6×10^{-8} m^2 s^{-3}, $N_0 = 0.125, 0.25, 0.5, 1.0$, and 2.0×10^{-2} s^{-1}, $L_0 = L_D/2$, and $f = 10 \times 10^{-5}$ s^{-1} for pure CT, and $U_*^2 = 2.0 \times 10^{-4}$ m^2 s^{-2}, $B_f = 1.225$ and 2.45×10^{-8} m^2 s^{-3}, $N_0 = 1.0 \times 10^{-2}$ s^{-1}, $L_0 = L_D/2$, and $f = 5.0 \times 10^{-5}$ s^{-1} and $U_*^2 = 1.0 \times 10^{-4}$ m^2 s^{-2}, $B_f = 2.45, 4.9, 9.8$, and 19.6×10^{-8} m^2 s^{-3}, $N_0 = 0.25 \times 10^{-2}$ s^{-1}, $L_0 = L_D/2$, and $f = 5.0 \times 10^{-5}$ s^{-1} for coexisting ST and CT. A total of 6, 15, and 6 simulations with higher resolution for pure ST, pure CT, and coexisting ST and CT, respectively, was compared to the original simulations with the corresponding parameters to the high-resolution simulations.

Figure S1 shows the Ozmidov scale ($L_{OZM} = \varepsilon^{1/2}/N^{3/2}$) normalized by the grid spacing (Δ) (L_{OZM}/Δ) as a function of Ro ($\equiv U_*/fL_{MLD}$) for pure ST simulations, $\mathrm{Ro_b}$ ($\equiv W_*/fL_{MLD}$) for pure CT simulations, and P_b^C/P_b^S for coexisting ST and CT simulations. Figure S1 also shows scatter plots of Ro and P_b^S normalized by U_*^3/L_{MLD} for pure ST simulations (cf. Fig. 2c), $\mathrm{Ro_b}$ and P_b^C normalized by B_f for pure CT simulations (cf. Fig. 4c), and P_b^C/P_b^S and P_b normalized by $P_b^S + P_b^C$ for coexisting ST and CT simulations (cf. Fig. 5c). In all the high-resolution simulations, L_{OZM} is greater than Δ, suggesting turbulent structures are well reproduced (Figs. S1a-c). In the several original simulations, on the other hand, L_{OZM} is smaller than Δ. In the original simulations, however, the normalized P_b was similar to that in the high-resolution simulations although it was slightly underestimated (Figs. S1d-f). Figure S1f also shows the $P_b/(P_b^S + P_b^C) \cong 0.6$ at $P_b^C/P_b^S \sim 10$ and $P_b/(P_b^S + P_b^C) \cong 1.0 - 1.2$ at the smallest and largest P_b^C/P_b^Ss in high-resolution simulations, indicating that the nonlinear interaction between ST and CT decreases P_b in the high-resolution simulations as well as in the original simulations as described in Results section. These suggest the present SGS parameterization described in Methods section works fine in our original LESs and our results with original simulations are robust.

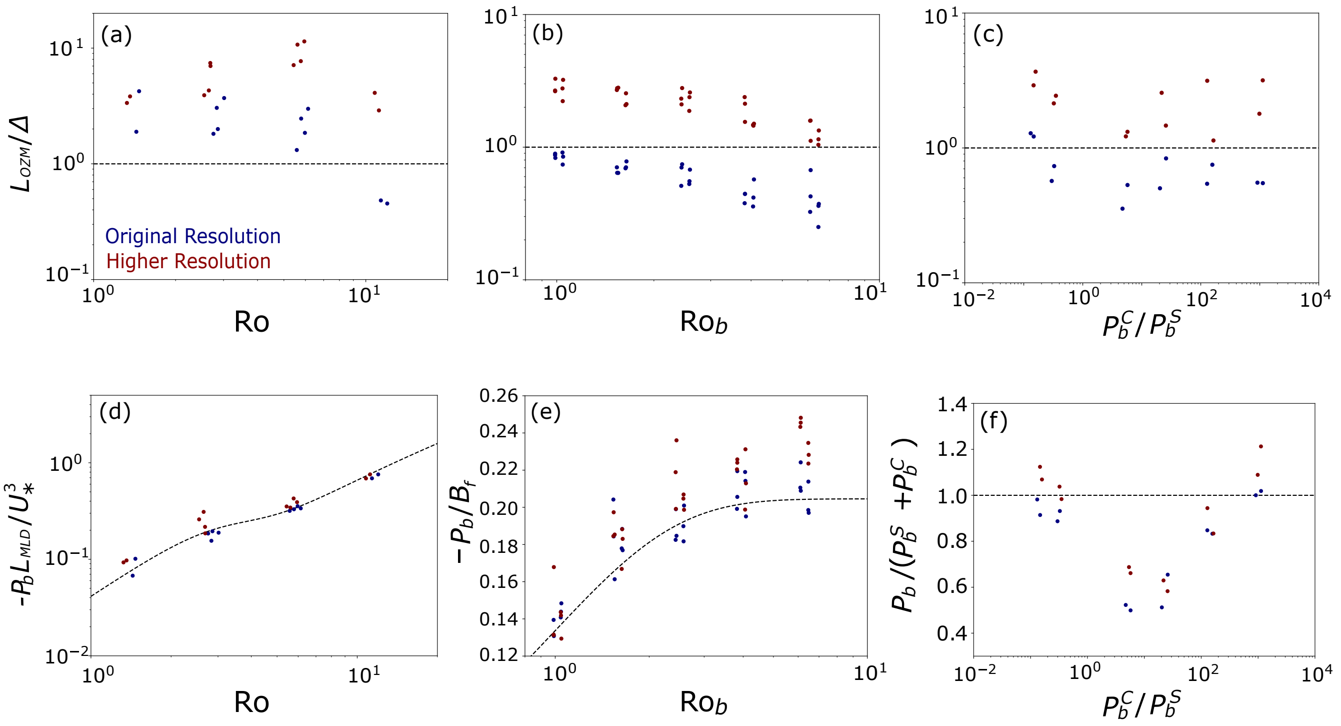

Figure S1. Normalized Ozmidov scale (L_{OZM}/Δ) as a function of (a) Ro for pure ST simulations, (b) $\mathrm{Ro_b}$ for pure CT simulations, and (c) P_b^C/P_b^S for coexisting ST and CT simulations. Scatter plots of (d) Ro and $-P_bL_{MLD}U_*^3$ for pure ST simulations, (e) $\mathrm{Ro_b}$ and $-P_b/B_f$ for pure CT simulations, and (f) P_b^C/P_b^S and $P_b/(P_b^S+P_b^C)$ for coexisting ST and CT simulations. Variables are averaged over $2.5 < t/T_f < 3.5$ and $4.0 < t/T_f < 5.0$. Colors represent the resolution. Dashed lines in (d) and (e) are the scalings derived in this study [Eqs. (7) and (10)].
